# Assessing Patient Satisfaction Following Otoplasty: A Social Media Analysis

**DOI:** 10.3390/cmtr19010011

**Published:** 2026-02-06

**Authors:** Shervin Eskandari, Gianluca Ramirez, Benjamin Aderinwale, Robel Yohannes, David Zabel

**Affiliations:** 1 Medical College of Georgia, Augusta University, Augusta, GA 30912, USA; 2 Department of Plastic and Reconstructive Surgery, Medical College of Georgia, Augusta University, Augusta, GA 30912, USA

**Keywords:** otoplasty, ear surgery, social media, RealSelf, facial plastic surgery, aesthetic surgery

## Abstract

Background: Otoplasty is a commonly performed cosmetic ear procedure, yet patient-reported outcome data remain limited. This study analyzes otoplasty reviews on RealSelf, a widely used aesthetic review platform that provides insight into patient experiences and outcomes. Methods: A retrospective review of otoplasty-related posts on RealSelf from January 2009 to March 2025 was performed. Reviews were manually screened and coded independently by reviewers. Extracted variables included demographics, procedure location, surgeon specialty, anesthesia type, postoperative pain, satisfaction rating (“Worth It,” “Not Worth It,” “Not Sure”), cost, motivations for surgery, and reasons for choosing the surgeon. Results: A total of 615 reviews met inclusion criteria, and 90.7% rated the procedure as “Worth It.” Protruding ears were the most common motivation for surgery (55.1%), followed by ear asymmetry (17.0%). Surgeon selection was most influenced by the consultation experience (34.4%), credentials (24.8%), and online reviews (21.6%). Positive surgeon comments emphasized comfort (32.3%), personality (27.9%), and communication (25.1%). Satisfaction was significantly associated with postoperative pain level (*p* < 0.001) and improved confidence after surgery (*p* = 0.032), but not with age, gender, procedure location, anesthesia type, or cost. Improved confidence (38.5%), enhanced ear shape (27.8%), and natural-appearing results (17.4%) were the most frequently cited reasons for being satisfied with otoplasty. Conclusion: Patient-reported satisfaction with otoplasty on RealSelf is high and is associated with favorable aesthetic results, improved self-confidence, and positive surgeon–patient interactions. In this cohort, effective communication, realistic expectation setting, and postoperative pain management were central to optimizing the patient experience.

## 1. Introduction

Otoplasty is a commonly performed cosmetic ear procedure used to correct prominent ears, asymmetry, congenital deformities, and acquired defects [1,2]. In 2023, more than 37,000 ear surgeries were performed, making it one of the most frequently performed aesthetic procedures [3]. Because many individuals have often lived with ear-related self-consciousness since childhood, the decision to undergo otoplasty is often strongly influenced by psychosocial factors [4,5].

Over the past decade, social media has become a major source of information for individuals considering aesthetic procedures [6]. Platforms such as Instagram, TikTok, and RealSelf allow patients to access before-and-after photos, surgeon reviews, and real-time accounts of postoperative recovery [7,8,9,10]. This growth has reshaped how patients research surgeons and gauge expected results to evaluate whether a procedure aligns with their goals [11,12]. For aesthetic medicine specifically, RealSelf has emerged as one of the most influential online communities [13], offering users the ability to post reviews, document their experiences, and rate their outcomes as “Worth It,” “Not Worth It,” or “Not Sure.” Unlike generic rating sites, RealSelf provides procedure-specific insights and surgeon-specific feedback, making it a valuable resource for studying patient perspectives.

While prior studies have broadly examined social media reviews in relation to a variety of plastic surgery procedures [14,15,16,17,18,19], RealSelf reviews specific to otoplasty have not been studied. Understanding how otoplasty patients use social media, how they choose their surgeons, and what factors shape their satisfaction may help guide patient counseling and improve the overall experience. Thus, the objective of this study was to provide a comprehensive analysis of otoplasty reviews on RealSelf, allowing insight into the role of social media in shaping expectations and evaluating outcomes for this procedure.

## 2. Methods

### 2.1. Study Design

A retrospective review of publicly available otoplasty reviews on RealSelf [20], was conducted to evaluate patient-reported experiences associated with the procedure. Using the search term “otoplasty,” all reviews published between January 2009 and March 2025 were identified, and all reviews that included a post describing an otoplasty experience were included. Reviews unrelated to otoplasty, advertisements, or duplicate entries were excluded.

### 2.2. Data Extraction

Each review was manually examined and coded independently by the authors. Extracted variables included demographic information (age, gender, location), procedure characteristics (surgeon specialty, anesthesia type, cost, postoperative pain), and satisfaction rating (“Worth It,” “Not Worth It,” “Not Sure”). Additional narrative-based variables included motivations for pursuing surgery, factors influencing surgeon selection, reasons for liking or disliking the surgeon, and reasons for liking or disliking the surgical outcome.

Narrative-based variables were coded when reviewers explicitly described their motivations or experiences. Motivations for pursuing surgery included protrusion, asymmetry, macrotia, trauma, malignancy, keloid, or dissatisfaction with ear shape. Reasons for selecting a surgeon included consultation experience, surgeon credentials, online reviews, personal or physician referrals, pricing structure, or surgical technique. Reasons for liking a surgeon included making the patient feel comfortable, personality, bedside manner, clarity of communication, responsiveness to concerns, postoperative availability, or perceived surgical skill and artistry. Reasons for liking a surgical outcome included improved confidence, enhanced ear shape or symmetry, natural appearance, well-hidden scars, or perceived value relative to cost. Explicit negative statements were also categorized as unfavorable outcomes.

All coded variables were compared between authors, and discrepancies were resolved through group consensus. Cases without clear narrative information for a given variable were left unclassified. Additionally, several variables were reported by only a subset of reviewers. Accordingly, valid frequencies and percentages are presented, excluding missing data.

### 2.3. Statistical Analysis

All data were compiled and analyzed using SPSS Statistics v29.0.0.0.0 (IBM, Armonk, NY, USA). Descriptive statistics were calculated for all variables. Associations between categorical variables and satisfaction rating were assessed using chi-square tests, while differences in continuous variables, such as cost, were evaluated using the Mann–Whitney U test. Statistical significance was defined as *p* < 0.05.

## 3. Results

### 3.1. Patient Demographics

A total of 615 otoplasty reviews were included in this study. Gender was reported in 189 reviewers, of whom 57.7% were female (*n* = 109) and 42.3% were male (*n* = 80). The mean age was 28.9 years (SD 15.1; *n* = 76), with 26.3% between 18–24 (*n* = 20) and 31.6% between 25–34. Among the 610 reviews reporting location, most originated from the United States (60.5%), with California (22.0%), New Jersey (11.9%), and Florida (8.7%) being the most frequently represented states. International reviews accounted for 39.5% of the cohort, primarily from England (46.5%), Canada (13.7%), and Australia (10.8%). Further details regarding demographic variables can be found in Table 1.

### 3.2. Procedure Specifics

Surgeon specialty was reported for 599 reviewers. Most procedures were performed by plastic surgeons (94.3%, *n* = 565), followed by ENT surgeons (5.2%, *n* = 31) and OMFS specialists (0.5%, *n* = 3). Anesthesia method was reported for 70 cases, with 81.4% receiving local anesthesia (*n* = 57) and 18.6% receiving general anesthesia (*n* = 13). Postoperative pain ratings were available for 115 reviewers: 13.0% reported no pain (*n* = 15), 34.8% minimal pain (*n* = 40), 21.7% moderate pain (*n* = 25), and 30.4% severe pain (*n* = 35). Among the 507 reviewers providing satisfaction ratings, 90.7% (*n* = 460) rated the procedure as “Worth It,” 4.1% (*n* = 21) as “Not Worth It,” and 5.1% (*n* = 26) as “Not Sure.” The average reported cost was $4589 (SD $3551; *n* = 223), and the mean recovery time was 8.1 days (SD 10.1; *n* = 15). Additional procedural information is summarized in Table 2. 

### 3.3. Reasons for Pursuing Otoplasty

A total of 236 reviewers provided reasons for undergoing otoplasty, with the most common reason being protruding ears (55.1%, *n* = 130). This was followed by auricular asymmetry (17.0%, *n* = 40) and macrotia (9.7%, *n* = 23). Less frequently reported indications included post-traumatic deformity (5.9%, *n* = 14), malignancy-related reconstruction (5.1%, *n* = 12), misshapen ears (4.7%, *n* = 11), and keloid excision (2.5%, *n* = 6). A breakdown of these motivations is presented in Table 3.

### 3.4. Reasons for Choosing Surgeon

Among 124 reviewers who reported factors influencing surgeon selection, the most common reason was a positive experience during the initial consultation (34.4%, *n* = 43). This was followed by physician credentials (24.8%, *n* = 31), online reviews (21.6%, *n* = 27), physician referral (5.6%, *n* = 7), and referrals from previous patients (5.6%, *n* = 7). Less frequently cited factors included pricing structure (2.4%, *n* = 3) and technique (1.6%, *n* = 2). Further details regarding surgeon selection criteria are provided in Table 4.

### 3.5. Reasons for Liking Surgeon

Among 362 reviewers who described positive aspects of their surgeon, the most frequently cited reason was feeling comfortable with the surgeon (32.3%, *n* = 117). Additional commonly reported factors included the surgeon’s personality (27.9%, *n* = 101), good communication (25.1%, *n* = 91), and perceived surgical skill or artistry (13.5%, *n* = 49). A small subset of reviewers (1.1%, *n* = 4) reported regretting their choice of surgeon. These preferences are detailed in Table 5.

### 3.6. Reasons for Liking Surgical Outcome

Among 299 reviewers who reported specific aspects of their outcome, 38.5% (*n* = 115) cited improved confidence. Additional common themes included improved ear shape (27.8%, *n* = 83), achieving a natural result (17.4%, *n* = 52), well-hidden scars (3.7%, *n* = 11), and favorable cost (0.3%, *n* = 1). A subset of reviewers (12.4%, *n* = 37) specifically reported that they did not like their surgical outcome. Descriptions of outcome-related feedback can be found in Table 6.

### 3.7. Comparison of Worth It, Not Worth It, and Not Sure Reviews

Cost did not differ significantly between satisfied and dissatisfied reviewers, with “Worth It” reviews costing an average (SD) of $4664 ($3756) compared to $4198 ($2210) among those who rated their procedure as “Not Worth It” or “Not Sure” (*p* = 0.699). The mean cost of otoplasty was significantly higher for U.S-based procedures compared to international procedures [Mean (SD) = $4845 ($3857) vs. $3858 ($2430), *p* = 0.005]. Furthermore, patients reporting improved confidence after surgery were significantly more likely to rate otoplasty as “worth it” compared with those who did not (97.1% vs. 75.8%, OR 10.8, 95% CI 3.2–36.3, *p* = 0.032). Patients reporting minimal or no pain after surgery were also significantly more likely to rate otoplasty as “worth it” compared with those who reported moderate or severe pain (98.0% vs. 70.1%, OR 20.6, 95% CI 2.7–158, *p* < 0.001). Additionally, there were no associations between satisfaction and age group (*p* = 0.652), gender (*p* = 0.106), procedure location (*p* = 0.437), or anesthesia type (*p* = 0.582). 

## 4. Discussion

The rise of the digital era has resulted in online patient-generated reviews becoming an important resource for both prospective patients and surgeons. Social media now serves as a major venue for sharing healthcare experiences, and RealSelf specifically is one of the largest platforms dedicated to aesthetic procedures, allowing users to describe their surgical journey and rate whether their outcome was “Worth It” or “Not Worth It.” With more than two million posted reviews and roughly ten million monthly visitors, the site represents a substantial repository of patient perspectives [13]. These reviews not only guide individuals considering treatment but also offer physicians insight into patient priorities and drivers of satisfaction. Thus, our study aimed to examine otoplasty-related patient reviews on RealSelf to identify experiential factors associated with the procedure.

Our findings demonstrated an overall positive sentiment towards otoplasty results, with 90.7% of reviewers rating their procedure as “Worth It.” This satisfaction rate is comparable to those reported for other facial cosmetic procedures on RealSelf, including rhytidectomy (91.9%), blepharoplasty (93.5%), and rhinoplasty (93.8%) [17,18,19]. Additionally, our analysis showed that patient satisfaction was closely tied to the patient’s interpersonal experience with their surgeon, with positive reviews often emphasizing how comfortable, understood, or reassured patients felt during their interactions with their surgeon. This focus on surgeon demeanor reflects prior findings showing that patients’ satisfaction with aesthetic procedures is closely tied to how they perceive their provider and how well they feel their concerns are addressed [21,22,23]. Thus, achieving optimal satisfaction after cosmetic surgery may involve factors beyond aesthetic results alone, and surgeons can consider focusing on trust-building efforts that support patients in feeling valued and cared for throughout the treatment journey.

Improved confidence and aesthetic outcomes were also top factors for patient satisfaction in our study. The importance of the aesthetic result was further reflected in the reasons cited for dissatisfaction, as patients who left negative reviews often described concerns such as visible scarring or an unnatural result, underscoring the importance of achieving an outcome that aligns with patient expectations. Together, these observations reinforce that aesthetic surgery patients, understandably, place substantial weight on the final cosmetic result [24,25], and that unmet aesthetic expectations can overshadow other aspects of care. This underscores the importance of clear preoperative counseling and aligning realistic surgical goals with patient priorities.

Our analysis also indicated that reviewers who reported minimal or no pain were significantly more likely to describe their otoplasty experience positively. This aligns with prior findings demonstrating that postoperative discomfort can meaningfully influence patient perception of the overall surgical experience [26,27]. Such experiences emphasize the importance of effective perioperative counseling and pain management strategies, which may contribute to higher satisfaction even when other aspects of recovery are challenging.

Patient-reported satisfaction has long been recognized as a key indicator of surgeon performance, and understanding the elements that shape postoperative experiences can meaningfully guide surgical practice. Online reviews, both positive and negative, can play a role in how individuals choose surgeons and make decisions about elective procedures [28]. Prior work has even shown that favorable reviews on RealSelf can contribute to increased clinic volume [29], and that patient-generated comments and ratings on social media may carry more weight in shaping patients’ decision making than objective qualifications like years of experience [30]. Conversely, negative feedback has also been demonstrated to deter patients during the decision-making process [31,32]. For surgeons, these reviews offer insight into what patients value, highlighting aspects of care that are well received and areas where improvements could enhance the overall patient experience.

This study is not without its limitations. Because our dataset was derived from an online review platform, it is subject to selection bias, as individuals with particularly strong positive or negative experiences may be more inclined to share their outcomes publicly, and users of such platforms may not represent the broader otoplasty patient population. Reviews also varied considerably in level of detail, completeness, and the amount of demographic or procedural information provided, limiting the ability to perform standardized comparisons across all cases. The inherently subjective nature of patient-reported experiences introduces additional bias, as satisfaction may be influenced by preexisting self-perception, unrealistic expectations, or selective emphasis on certain aspects of the experience. Although RealSelf independently verifies reviews and restricts physicians from removing negative content, the possibility remains that some practices may disproportionately encourage satisfied patients to post reviews, thereby skewing the distribution of sentiments. Additionally, while reviews were independently coded by multiple authors with discrepancies resolved by consensus, formal inter-rater reliability statistics were not calculated, which may introduce subjectivity into the qualitative coding process. Furthermore, our analysis relied on patient-reported narratives rather than validated patient-reported outcome measures, and objective clinical details such as surgical technique, revision status, and standardized outcome assessments could not be independently verified. Despite these limitations, the large number of reviews and the rich qualitative information available offer valuable insight into how patients perceive their otoplasty results and which factors most strongly influence satisfaction.

## 5. Conclusions

This review of 615 RealSelf otoplasty posts demonstrates that patient satisfaction with otoplasty is high, with the majority of individuals reporting their procedure as “Worth It” and describing meaningful improvements in confidence and ear appearance. Patients most often pursued surgery due to protruding ears, selected surgeons based on interpersonal qualities and perceived expertise, and valued natural-appearing outcomes. Low postoperative pain emerged as a key factor associated with satisfaction, underscoring the importance of clear perioperative counseling and effective pain management strategies.

## Figures and Tables

**Table 1:** Reviewer Demographics.

Characteristic	*n* (%)
Gender	189 (100.0)
Female	109 (57.7)
Male	80 (42.3)
Age group	76 (100.0)
<18	14 (18.4)
18–24	20 (26.3)
25–34	24 (31.6)
35–54	13 (17.1)
55+	5 (6.6)
Procedure Location	610 (100.0)
Domestic	369 (60.5)
California	81 (22.0)
New Jersey	44 (11.9)
Florida	32 (8.7)
New York	31 (8.4)
Other U.S. states	181 (49.1)
International	241 (39.5)
England	112 (46.5)
Canada	33 (13.7)
Australia	26 (10.8)
Turkey	22 (9.1)
Other countries	48 (19.9)

**Table 2:** Procedure Characteristics. ENT = otolaryngology. OMFS = oral and maxillofacial surgery.

Characteristic	*n* (%)
Surgeon Specialty	599 (100.0)
Plastic Surgery	565 (94.3)
ENT	31 (5.2)
OMFS	3 (0.5)
Anesthesia Method	70 (100.0)
Local anesthesia	57 (81.4)
General anesthesia	13 (18.6)
Postoperative Pain	115 (100.0)
None	15 (13.0)
Minimal	40 (34.8)
Moderate	25 (21.7)
Severe	35 (30.4)
Procedure Cost	223 (100)
$0–1999	24 (10.8)
$2000–3999	77 (34.5)
$4000–5999	79 (35.4)
$6000–7999	30 (13.5)
$8000+	13 (5.8)
Worth It Rating	507 (100.0)
Worth It	460 (90.7)
Not Worth It	21 (4.1)
Not Sure	26 (5.1)

**Table 3:** Reasons for Pursuing Otoplasty.

Reason	*n* (%)
Protruding ears	130 (55.1)
Asymmetrical ears	40 (17.0)
Large ears	23 (9.7)
Trauma	14 (5.9)
Malignancy	12 (5.1)
Misshapen ears	11 (4.7)
Keloid	6 (2.5)
Total	236 (100.0)

**Table 4:** Reasons for Choosing Surgeon.

Reason	*n* (%)
Experience at initial consult	43 (34.4)
Physician credentials	31 (24.8)
Online reviews	27 (21.6)
Physician referral	7 (5.6)
Referral from previous patients	7 (5.6)
Regrets choosing surgeon	4 (3.2)
Pricing structure	3 (2.4)
Technique	2 (1.6)
Total	124 (100.0)

**Table 5:** Reasons for Liking Surgeon.

Reason for Liking Surgeon	*n* (%)
Made me feel comfortable	117 (32.3)
Liked surgeon personality	101 (27.9)
Good communication	91 (25.1)
Surgical skill or artistry	49 (13.5)
Regrets choosing surgeon	4 (1.1)
Total	362 (100.0)

**Table 6:** Reasons for Liking Surgical Outcome.

Reason for Liking Outcome	*n* (%)
Improved confidence	115 (38.5)
Ear shape improved	83 (27.8)
Natural result	52 (17.4)
Scars well hidden	11 (3.7)
Liked price	1 (0.3)
Did not like outcome	37 (12.4)
Total	299 (100.0)

## Data Availability

The datasets generated and/or analyzed during the current study are available upon reasonable request.
